# Distribution of Urocortins and Corticotropin-Releasing Factor Receptors in the Cardiovascular System

**DOI:** 10.1155/2012/395284

**Published:** 2012-05-17

**Authors:** Kazuhiro Takahashi

**Affiliations:** Departments of Endocrinology and Applied Medical Science, Tohoku University Graduate School of Medicine, 2-1 Seiryo-machi, Aoba-ku, Sendai, Miyagi 980-8575, Japan

## Abstract

Urocortins are human homologues of urotensin I, a fish corticotropin-releasing-factor- (CRF-) like peptide secreted from the urophysis. There are three urocortins: urocortin 1, urocortin 2, and urocortin 3 in mammals. We have shown that urocortin 1 and urocortin 3 are endogenously synthesized in the myocardial cells of human heart and may act on CRF type 2 receptor (CRFR2) expressed in the heart. Expression levels of urocortin 1 in the heart and plasma urocortin 1 levels are elevated in patients with heart failure. Recent studies have shown that urocortins have various biological actions in the cardiovascular system, such as a vasodilator action, a positive inotropic action, a cardioprotective action against ischemia/reperfusion injury, and suppressive actions against the renin angiotensin system and the sympathetic nervous system. Urocortins and CRFR2 may therefore be a potential therapeutic target for cardiovascular diseases, such as congestive heart failure, hypertension, and myocardial infarction.

## 1. Introduction

 Actions of the corticotropin-releasing factor (CRF) are mediated by two types of G-protein coupled receptors: CRF type 1 receptor (CRFR1) [1] and CRF type 2 receptor (CRFR2) [2]. CRFR1 mediates ACTH responses to stress [3, 4], whereas CRFR2 mediates stress-coping responses including anxiolysis, anorexia, vasodilatation, a positive inotropic action on myocardium, and dearousal [5–7]. Mice deficient for CRFR2 show anxiety-like behavior, are hypersensitive to stress, and have elevated blood pressure [5–7]. CRF receptors, particularly CRFR2, are expressed in the heart and systemic vasculature [2, 8–11]. However, CRF expression is very low or undetectable in the heart and blood vessels, and circulating levels of CRF in plasma are also very low. Endogenous ligands for CRFR2 expressed in the heart and systemic vasculature had been unknown for a long time. This paper is an overview of our current understanding of the expression and function of CRF receptors and their ligands in the cardiovascular system.

## 2. Discovery of Urocortins

 Urotensins are peptide hormones secreted from fish urophysis, the neuroendocrine organ located in the caudal spinal cord [12, 13]. Urotensin I was considered to be a CRF-like fish peptide, whereas urotensin II was a somatostatin-like fish peptide. Urocortin 1 (Ucn 1) was discovered from rat brain as a mammalian homologue of fish urotensin I [14]. Ucn1 binds to both CRFR1 and CRFR2 with similar affinities. Human Ucn 1 has 63% identity with fish urotensin I and 43% identity with CRF at the amino acid level.

 Furthermore, Ucn 2 (stresscopin-related peptide, SRP) and Ucn 3 (stresscopin, SCP) were discovered by searching the public genome databases and shown to be specific agonists for CRFR2 [15–17]. Ucn 2/SRP [15, 17] and Ucn 3/SCP [16, 17] were discovered by two independent research groups, which interpreted post-translational processing sites of the precursor proteins differently. The reported amino acid sequences of the peptides were therefore slightly different between Ucn 2 and SRP and between Ucn 3 and SCP. Human Ucn 2 is a 38-amino-acid peptide that corresponds to the sequence 6–43 of human SRP, a 43-amino-acid peptide. Human Ucn 3 is a 38-amino-acid peptide that corresponds to the sequence 3–40 of human SCP, a 40-amino-acid peptide. Ucn 2 and Ucn 3 have about 20–40% homology with CRF and Ucn 1. The homology between Ucn 2 and Ucn 3 was about 40%.

 Thus, the CRF family consists of CRF, Ucn 1, Ucn 2 (SRP), and Ucn 3 (SCP) as well as fish urotensin I and frog sauvagine. Urocortins (Ucns) were shown to be expressed in various tissues and cells of the human, such as brain, pituitary, gastrointestinal tract, ovary, placenta, synovial tissue, lymphocytes, and skin [16–28].

## 3. Expression of Urocortin 1 and 3 and CRF Receptors in Cardiovascular Tissues

 We have shown expression of Ucn 1, Ucn 3, and CRF receptors in the human heart obtained at autopsy [29, 30].

**Figure 1:**
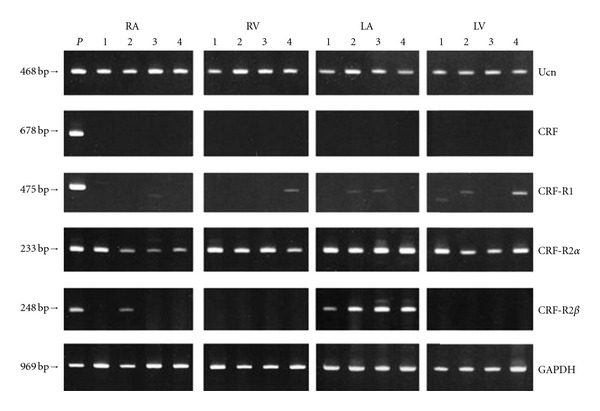
RT-PCR analysis for urocortin 1 (Ucn), CRF, CRF type1 receptor (CRF-R1), CRF type 2*α* receptor (CRF-R2*α*), and CRF type 2*β* receptor (CRF-R2*β*) mRNAs in four human hearts (patients 1–4) in the four constituent chambers (RA, right atrium; RV, right ventricle; LA, left atrium; LV, left ventricle). Total RNA from placenta, pituitary gland, hypothalamus, and left atrium were used as positive controls for urocortin 1 and CRF, CRF-R1, -R2*α*, and -R2*β*, respectively. As a negative control for RT-PCR, RT was performed on total RNA samples in the absence of reverse transcriptase enzyme (data not shown). The bottom panel shows GAPDH employed as an internal control. P: Positive control (reproduced from [29] with kind permission from the Endocrine Society. *Copyright 2002, The Endocrine Society*).

**Figure 2:**
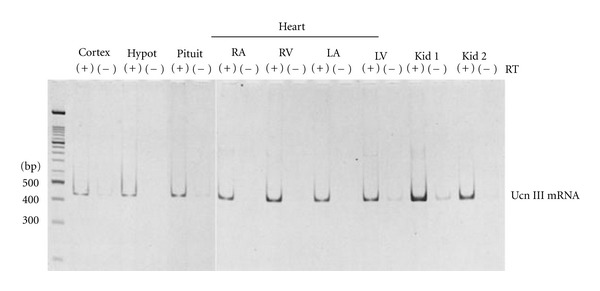
RT-PCR of urocortin 3 (Ucn III) mRNA in brain, pituitary, heart, and kidneys. Cortex: cerebral cortex; Hypot: hypothalamus; Pitui: pituitary; RA: right atrium; RV: right ventricle; LA: left atrium; LV: left ventricle; Kid 1 and Kid 2: kidneys. RT(−) indicates negative controls (samples without reverse-transcriptase treatment). A 469 base-pair DNA fragment corresponding to 5/473 of the human urocortin 3 gene (GeneBank accession number AF361943) was amplified by RT-PCR (reproduced from [30] with kind permission from the Endocrine Society. *Copyright 2004, The Endocrine Society*).

 First, reverse-transcriptase- (RT-) PCR analysis demonstrated that Ucn 1 mRNA was expressed in the right atrium, right ventricle, left atrium, and left ventricle in all cases studied (Figure 1) [29]. However, CRF mRNA was not detected in any of these samples. CRFR2*α* mRNA was expressed in four chambers of all cases studied. A weak band for CRFR1 mRNA was detected in the left atria of patients 2 and 3, in the left ventricles of patients 2 and 4, and in the right ventricle of patient 4. Although CRFR1 may be present in the heart, CRFR1 mRNA was detected in the heart of limited cases due to the low expression levels in most cases. CRFR2*β* mRNA expression was observed in the left atrium in all cases and in the right atrium of only one out of four cases studied (patient 2). Thus, the major CRF receptor subtype expressed in all four chambers of the human heart was CRFR2*α*. Moreover, RT-PCR analysis showed expression of Ucn 3 mRNA in the cerebral cortex, hypothalamus, pituitary, ventricles and atria of heart, and kidney (Figure 2) [30]. RNA samples without reverse-transcriptase treatment gave no band or very weak bands, indicating that effects of genomic RNA contamination into the RNA samples were negligible.

**Figure 3:**
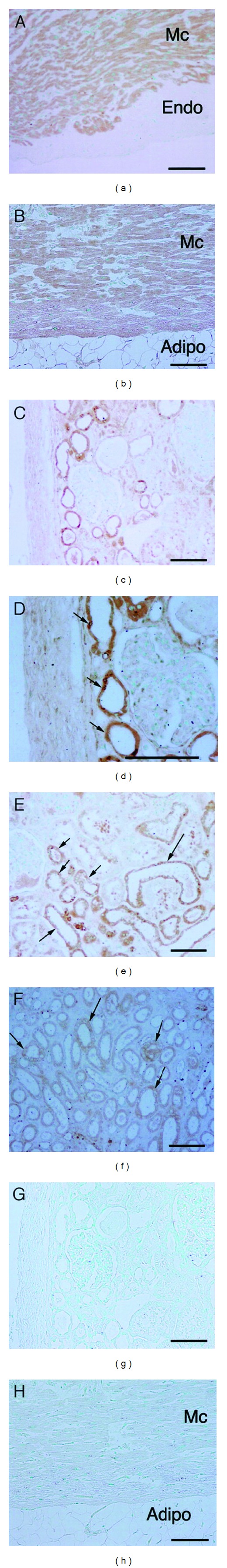
Immunocytochemistry of urocortin 3 in human heart and kidney. (a) and (b) Myocardium (Mc) was positively stained for uroccortin 3, whereas endocardium (Endo) and pericardial adipocytes (Adipo) were not. (c)–(e) Renal tubular cells in the renal cortex, particularly distal tubules, were strongly stained for urocortin 3 (shown by arrows in (d)); (d) a higher magnification of (c). (f) Renal tubular cells in the renal medulla were weakly stained for urocortin 3 (arrows). Typical urocortin 3-positive renal tubules were indicated by arrows (d)–(f). (g) and (h) Negative controls of kidney and heart using normal rabbit serum (1 : 1000). (g) A serial section of kidney (c); (h) a serial section of heart (b). Bars, 100 *μ*m (reproduced from [30] with kind permission from the Endocrine Society, *Copyright 2004, The Endocrine Society*).

 Immunocytochemistry showed that Ucn 1 and CRF receptor immunoreactivities were detected in the great majority of myocardia of human heart [29]. Positive immunostaining of Ucn 3 was also observed in the myocardium [30] (Figures 3(a) and 3(b)). In addition to the heart, Ucn 3 was immunostained in the proximal and distal tubules of the kidney (Figures 3(c)–3(f)) [30]. Particularly, strong immunostaining was observed in the distal tubules of the renal cortex (Figures 3(c)–3(e)). Renal tubules in the renal medulla were weakly stained for Ucn 3 (Figure 3(f)). Negative controls using normal rabbit serum instead of the Ucn 3 antiserum showed no positive immunostaining (Figures 3(g) and 3(h)). The absorption of the antiserum with synthetic Ucn 3 (10 nmol Ucn 3/mL diluted antiserum) abolished positive immunostaining (data not shown).

 Specific radioimmunoassay for Ucn 1 and Ucn 3 could detect respective immunoreactivity in the human heart. The antisera against Ucn 1 and Ucn 3 were raised in rabbits [18, 30]. The same antisera used in immunocytochemistry were used in radioimmunoassay, respectively. The cross-reactivity of the Ucn 1 antiserum was less than 0.001% with CRF, Ucn 2, Ucn 3, atrial natriuretic peptide (ANP), and other cardiovascular peptides. The radioimmunoassay of Ucn 1 could detect changes of 10 fmol/tube from zero at 95% confidence with duplicate tubes [18]. The cross reactivity of the Ucn 3 antiserum was less than 0.001% with CRF, Ucn 1, Ucn 2, ANP, and other cardiovascular peptides. The radioimmunoassay of Ucn 3 could detect changes of 4.7 fmol/tube from zero at 95% confidence with duplicate tubes [30].

 The highest concentrations of immunoreactive- (IR-) Ucn 1 were found in the left ventricle (1.90 ± 0.5 pmol/g wet weight, mean ± SEM), followed by the right ventricle (1.26 ± 0.3 pmol/g wet weight), the left atrium (1.21 ± 0.2 pmol/g wet weight), and the right atrium (0.92 ± 0.1 pmol/g wet weight) [29]. IR-Ucn 3 was detected in the human heart tissues (0.74–1.15 pmol/g wet weight) and kidney tissues (1.21 ± 0.30 pmol/g wet weight) [30]. IR-Ucn 3 was present in both ventricles and atria of hearts, and no significant difference was noted among them. The levels of IR-Ucn 3 in the heart were comparable with the levels found in the human brain tissues. Thus, both Ucn 1 and Ucn 3 are diffusely distributed in four chambers of human hearts, and no apparent difference was noted in the distribution between Ucn 1 and Ucn 3 in human heart. It has not been clarified yet whether there are any differences of the physiological significance between Ucn 1 and Ucn 3 in the heart.

 Ucn 1 mRNA expression was shown in cultured rat cardiac myocytes and nonmyocytes [31]. IR-Ucn 1 detected in the human heart tissues may therefore be derived from both cardiomyocytes and other types of cells, such as fibroblasts and vascular endothelial and smooth muscle cells. Actually, Ucn 1, Ucn 2, and Ucn 3 mRNAs were all detected by RT-PCR in cultured human umbilical vein endothelial cells [32]. Immunocytochemical studies showed that Ucn 1 was expressed in myometrial vascular smooth muscle cells during pregnancy [33], and Ucn 2, but not Ucn 3 was expressed in vascular endothelial cells of placenta [34]. Ucns produced in the vasculature may also regulate vascular tone in a paracrine or autocrine fashion.

 IR-Ucn 1 and IR-Ucn 3 were shown to be present in human plasma (Ucn 1, 16.6 ± 5.5 pg/mL in 5 men and 12.8 ± 1.9 pg/mL in 6 nonpregnant women; Ucn 3, 51.8 ± 16.0 pmol/L in 5 men, mean ± SEM) [35, 36], but the levels were too low to exert biological actions on myocardium or vascular tissues as circulating hormones. Ucn 1 and Ucn 3 secreted from the heart are therefore likely to act on CRFR2 expressed in the myocardium as autocrine or paracrine factors. Wiley and Davenport showed that CRFR2 is highly expressed in human heart using radioligand binding techniques and autoradiographic studies [36]. Binding studies using [^125^I] antisauvagine 30 showed the presence of high affinity of CRFR2 receptor in human left ventricle with a Kd of 0.21 ± 0.03 nM.

 By contrast, the predicted amino acid sequence of human Ucn 2 (SRP) precursor lacks a consensus proteolytic cleavage site that would allow for C-terminal processing of the peptide [15]. It has not been clarified whether Ucn 2 peptide is present in human tissues, although Ucn 2 mRNA and Ucn 2-like immunoreactivity were detected in human tissues by RT-PCR and immunocytochemistry, respectively [34, 37].

## 4. Expression of Urocortins and CRF Type 2 Receptor in the Heart under Pathological Conditions

 Expression of Ucn 1 was shown to be elevated in the heart tissues obtained from patients with heart diseases [31, 38]. Nishikimi et al. performed immunocytochemistry of Ucn 1 in heart tissues obtained at autopsy from 5 patients without cardiovascular diseases and 5 patients with dilated cardiomyopathy, or surgical samples of a Batista operation in 4 patients with dilated cardiomyopathy, and showed that Ucn 1 immunoreactivity was more intense in the myocytes of failing heart than that in myocytes of the normal heart [31]. Ikeda et al. studied Ucn 1 expression in the diseased heart (12 cases of hypertrophic cardiomyopathy and 42 cases of dilated cardiomyopathy) by immunocytochemistry using endomyocardial biopsy specimens and showed that Ucn 1 was expressed more abundantly in the diseased heart, especially in hypertrophic cardiomyopathy and dilated cardiomyopathy, than in the normal heart [38].

 Furthermore, plasma levels of Ucn 1 are elevated in patients with chronic heart failure [39–41]. Ng et al. studied plasma levels of Ucn 1 in 119 patients with heart failure and 212 age- and gender-matched controls and showed elevated plasma Ucn 1 levels in heart failure patients (the mean Ucn 1 levels; normal males 19.5 pmol/L, heart failure male 50.2 pmol/L, normal females 14.2 pmol/L, heart failure females 21.8 pmol/L) [39]. The Ucn 1 levels fell with increasing age and with increasing New York Heart Association (NYHA) functional class. Wright et al. studied plasma Ucn 1 levels in 74 patients with chronic heart failure and 225 healthy subjects and found significant elevation of plasma Ucn 1 levels in patients with chronic heart failure (11.1 ± 3.2 pmol/L, mean ± SD), compared with healthy subjects (7.2 ± 2.9 pmol/L) [40]. In contrast to the report by Ng et al. [39], plasma Ucn 1 levels showed stepwise increase in proportion to NYHA functional classes in patients with heart failure. Plasma Ucn 1 levels were related to left ventricular dimensions and function assessed by echocardiography, such as left ventricular end-diastolic dimension, left ventricular end-systolic dimension, fractional shortening, and left ventricular ejection fraction. Furthermore, there were significant positive relationships between plasma Ucn 1 levels and levels of other cardiovascular hormones, including B-type natriuretic peptide (BNP), N-terminal proBNP, adrenomedullin, and endothelin-1. However, the elevation of plasma Ucn 1 levels was not so marked as that of these cardiovascular hormones in patients with heart failure. Gruson et al. also studied plasma Ucn 1 levels in 42 fully treated heart failure patients and 20 healthy age- and gender-matched subjects and found elevated Ucn 1 levels in heart failure patients (mean; 88 pmol/L) compared with control (46 pmol/L) [41]. Plasma Ucn 1 levels showed stepwise increase in proportion to NYHA functional classes in patients with heart failure, consistent with the report by Wright et al. [40], but were not significantly correlated with plasma levels of N-terminal proBNP, N-terminal proatrial natriuretic peptide (proANP), or big endothelin-1.

 Elevated plasma Ucn 1 in heart failure patients may be mainly derived from diseased heart. However, the plasma levels may be too low to exert biological actions on myocardium or vascular tissues as mentioned in the previous section. Ucn 1 in the diseased heart may therefore act locally on the myocardium as an autocrine or paracrine factor and affect the myocardial function in heart failure. CRFR2*β* mRNA expression was remarkably depressed in the left ventricle of DOCA-salt spontaneously hypertensive rats (SHRs) compared with control Wistar-Kyoto (WKY) rats, whereas Ucn 1 mRNA expression was elevated in DOCA-salt SHR [31]. CRFR2*β* mRNA expression in the heart was decreased by lipopolysaccharide, corticosterone, and physical restraint in rats [42]. Moreover, CRFR2*β* mRNA expression in A7R5 aortic smooth muscle cells was decreased by inflammatory cytokines, interleukin- (IL-) 1*β*, IL-6, and tumor necrosis factor *α* (TNF-*α*) [42]. Coste et al. showed that systemic administration of IL-1*α* and TNF-*α* downregulated CRFR2 expression in the mouse heart whereas Ucn 1 decreased CRFR2 expression in cultured cardiomyocytes [43]. They speculated that inflammatory mediators may increase expression of Ucn 1, which in turn downregulated CRFR2 expression in the heart.

 In addition to the cardiovascular and endocrine systems and endocrine tumors [19, 44–47], Ucn 1 is expressed in organs and cells of the immune system, such as lymphocytes [21, 25]. There is accumulating evidence that indicates the relation of Ucn 1 to inflammatory diseases. Expression of Ucn 1 is enhanced in the tissues with inflammatory diseases, such as rheumatoid arthritis and ulcerative colitis [24, 48]. Expression of Ucn 1 was upregulated in HL-1 cardiomyocytes by lipopolysaccharide, TNF-*α*, and oxidative stress [49]. Inflammatory mediators are important in the pathogenesis of chronic heart failure, contributing to cardiac remodeling and peripheral vascular disturbances [50]. Levels of inflammatory cytokines such as TNF-*α*, IL-1*β*, and IL-6 are elevated in plasma, circulating leukocytes, and tissues of failing myocardium in patients with chronic heart failure. Expression of Ucn 1 mRNA was also increased after heat shock at 42°C in cultured rat cardiac myocytes [51]. Ucn 1 expression may therefore be induced in cardiac myocytes by certain types of stress, such as inflammation and oxidative stress.

 Hypoxia induces expression of genes for various vasoactive peptides such as endothelin-1 [52] and adrenomedullin [53, 54], possibly via the transcriptional factor, hypoxia-inducible factor 1 (HIF-1). Hypoxia did not affect Ucn 1 secretion or mRNA expression in placental explants and primary cultures of human trophoblasts [55]. Hypoxia caused by ischemic heart disease is therefore not likely to explain the cause for the increased expression of Ucn 1 in diseased heart. By contrast, expression levels of Ucn 2 and Ucn 3 mRNA were increased by hypoxic stress in cultured rat neonatal cardiomyocytes [56]. Expression of Ucn 2 and Ucn 3 was induced by hypoxia in primary trophoblast cell cultures and explants via HIF-1 [57]. Moreover, Bühler et al. have shown that hypoxia induces human Ucn 2 expression via a specific hypoxia-responsive element in the 3′-flanking region of the human Ucn 2 gene [58]. Further studies are required to clarify whether expression of Ucn 2 or Ucn 3 is elevated in cardiac tissues with ischemic heart disease.

 There have been no reports on altered expression of Ucn 2 or Ucn 3 in diseased heart. The relation of Ucn 3 with inflammation may not be so marked as that of Ucn 1. In the colon mucosa, Ucn 3 was expressed in myenteric and submucosal nervous plexus, blood vessels, smooth muscle layers, and enterochromaffin cells but hardly detected lamina propria inflammatory cells in which Ucn 1 was abundantly expressed [59]. Moreover, no significant elevation of Ucn 3 expression was found in the colon mucosa from ulcerative colitis patients in contrast to Ucn 1 (Saruta and Takahashi, unpublished observations).

## 5. Potential Application of Urocortins in the Cardiovascular System

CRFR2 is abundantly expressed in the vasculature and all Ucns exert potent vasodilator actions [9, 36, 60–62]. In addition to vasodilator actions, Ucns have been shown to exert protective actions against cell death due to various stresses such as ischemia/reperfusion [51, 56, 63–65]. Actually, Ucn 1 was shown to enhance cardiac function during ischemia/reperfusion [64]. These actions of Ucns may be beneficial for the treatment of cardiovascular diseases, such as congestive heart failure, hypertension, and myocardial infarction.

All three Ucns prevented cardiomyocytes from ischemia/hypoxia-induced cell death and apoptosis via a MARK-dependent pathway [56, 63]. Ucn 2 and Ucn 3 protected cardiomyocytes from ischemia/reperfusion injury and reduce the percentage of infarct size in the murine heart via CRFR2 and ERK1/2-p42 and p-44 activation [64]. Cardiomyocytes isolated from CRFR2-null mice are less resistant to ischemia/reperfusion injury, compared with wild-type cardiomyocytes, indicating that CRFR2 is essential for the cardioprotective actions of Ucns. Furthermore, Ucns stimulated proliferation of cultured cardiomyocytes [66, 67]. Ucn 1 specifically induced enhanced expression of the mitochondrial K_ATP_ channel (ATP-sensitive inwardly rectifying potassium channel Kir 6.1) in cardiac myocytes [68]. The mitochondrial K_ATP_ channel is believed to be involved in the protective effects. Townsend et al. showed that Ucn 1 protected heart with reperfusion injury by inhibiting the mitochondrial permeability transition pore opening, possibly via a protein kinase C-mediated reduction in oxidative stress [69].

Barry et al. have recently reported that infusion of Ucn 1 or Ucn 2 before the onset of reperfusion resulted in the differential regulation of 66 and 141 genes, respectively, the majority of which have not been described previously [70]. Functional analysis demonstrated that Ucn-regulated genes are involved in a wide range of biological responses, including cell death, oxidative stress, and metabolism. In addition, both Ucn 1 and Ucn 2 modulated the expression of a host of genes involved in G-protein-coupled receptor signaling.

Pre- and postconditioning are powerful endogenous adaptive response of the organism whereby different stimuli enhance the tolerance against various types of stress. Cserepes et al. showed that preconditioning with Ucn 1 induced a powerful cell protective effect, which was comparable with that of adenosine and ischemia, using isolated neonatal rat ventricular myocytes subjected to ischemia and reperfusion [71]. Furthermore, postconditioning with Ucn 1 was more cardioprotective than ischemic postconditioning.

 The research group of Dr. Richards in New Zealand performed a series of experiments on cardiovascular effects of Ucns in sheep as well as in human subjects [72–80]. Administration of Ucn 1, Ucn 2, or Ucn 3 induced sustained reductions in cardiac preload and mean arterial pressure and improvements in cardiac input and renal function in sheep with heart failure induced by pacing [72–75]. Thus, Ucns have marked and beneficial hemodynamic, hormonal, and renal effects in experimental heart failure. These reports support the therapeutic potential of Ucns in heart failure. Furthermore, Ucns have inhibitory effects on the sympathetic nervous system and the renin-angiotensin system [76–79]. These inhibitory effects of Ucns may also be beneficial for the treatment of heart failure. Beneficial effects of Ucn 2 have been observed in human heart failure [80]. Intravenous administration of Ucn 2 (100 *μ*g Ucn 2 intravenously over 1 h) induced increases in cardiac output and left ventricular ejection fraction with falls in systemic vascular resistance and cardiac work in eight male patients with heart failure (left ventricular ejection fraction <40%, NYHA class II-III) [80].

Effects of Ucn 2 were also shown in mice and rats with heart failure. Bale et al. showed that Ucn 2 treatment augmented heart rate, exhibited potent inotropic and lusitropic actions on the left ventricle, and induced a downward shift of the diastolic pressure-volume relation in mice. Ucn 2 treatment also reduced systemic arterial pressure associated with a lowering of systemic vascular resistance [81]. These effects of Ucn 2 were not found in CRFR2-deficient mice, suggesting that these cardiovascular effects of Ucn 2 were mediated by CRFR2. Ucn 2 administration to cardiomyopathic mice (muscle-specific LIM protein-deficient mice) produced significant enhancement of inotropic and lusitropic effects on left ventricular function and improved cardiac output. Yang et al. showed that Ucn 2 had positive inotropic and lusitropic effects on mouse ventricular myocytes via activation of CRFR2 in a cAMP/protein kinase A- and Ca^2+^/calmodulin-dependent protein kinase II-dependent manner. However, this enhancement was accompanied by Ca^2+^-dependent arrhythmogenic effects mediated by protein kinase A and calmodulin-dependent protein kinase II [82]. By contrast, Meili-Butz et al. showed that administration of Ucn 2 rapidly improved left ventricular function and increased ventricular fibrillation threshold in failing hearts with increased propensity for ventricular arrhythmias, which were isolated from rats with hypertension-induced left ventricular hypertrophy and heart failure [83]. 

In addition to heart failure, Ucn 2 has been shown to be effective for hypertension. Dieteterle et al. studied effects of acute and chronic administration of Ucn 2 in hypertensive salt-sensitive Dahl rats and showed that Ucn 2 had immediate and sustained blood pressure-lowering effects without affecting heart rate [84]. Ucn 2 administration did not affect expression of Ucn 2 and CRFR2 in left ventricle and aorta of these rats. In addition to the direct effect on CRFR2 in the vasculature, Ucns may decrease blood pressure through the inhibitory effects on catecholamine synthesis and secretion in adrenal medulla and the sympathetic nervous system [37, 84]. Gu et al. showed that Ucn 2 lowered blood pressure and plasma catecholamine levels in chromogranin A-null mice, which had hyperadrenergic activity [84]. Dermitzaki et al. showed that CRFR1 agonists induced catecholamine secretion whereas CRFR2 agonists suppressed catecholamine secretion in dispersed human and rat adrenal chromaffin cells [37]. Moreover, activation of CRFR1 induced tyrosine hydroxylase, whereas activation of CRFR2 suppressed it in human chromaffin cells.

## 6. Perspectives

 Vasoconstrictor peptides, such as angiotensin II, endothelin-1, and urotensin II, promote the progression of cardiovascular and renal diseases and their complications [85–87]. Angiotensin II receptor antagonists and angiotensin-converting enzyme inhibitors are widely used for the treatment of cardiovascular diseases including congestive heart failure and hypertension [86]. By contrast, vasodilator peptides, such as ANP and adrenomedullin, have protective actions against tissue injuries caused by various stresses and may therefore be useful as drugs for certain cardiovascular and renal diseases [88–91]. Actually ANP is a very effective drug for acute heart failure [89].

 Ucns are cardiovascular peptides which are endogenously synthesized in the heart and vasculature and may play protective roles against various types of cardiovascular stress, such as hypoxia. Ucns and Ucn-related drugs may therefore have therapeutic potential for the treatment of cardiovascular diseases, such as congestive heart failure, hypertension, and myocardial infarction. Further studies are required to clarify whether Ucns have any merits as drugs for cardiovascular diseases over other drugs for cardiovascular diseases, including ANP, and which Ucn is the most effective for this purpose among three Ucns.
